# Comparative analysis of amino acid auxotrophies and peptidase profiles in non-dysbiotic and dysbiotic small intestinal microbiomes

**DOI:** 10.1016/j.csbj.2025.02.004

**Published:** 2025-02-12

**Authors:** Svenja Starke, Danielle M.M. Harris, Amandine Paulay, Konrad Aden, Silvio Waschina

**Affiliations:** a Institute of Human Nutrition and Food Science, Department of Nutriinformatics, Kiel University, Kiel, 24118, Germany; b Institute of Clinical Molecular Biology, Kiel University, Rosalind-Franklin-Straße 12, Kiel, 24118, Germany; c Department of Internal Medicine I, University Hospital Schleswig-Holstein, Campus Kiel, Kiel, 24105, Germany; d Université Paris-Saclay, INRAE, AgroParisTech, Micalis Institute, Jouy-en-Josas 78350, France; e Université Paris-Saclay, INRAE, MaIAGE, Jouy-en-Josas 78350, France; f Biomathematica, Ajaccio 20000, France

**Keywords:** Auxotrophies, Small intestinal microbiome, SIBO, Peptidases, Amino acids

## Abstract

Small Intestinal Bacterial Overgrowth (SIBO) is linked to various diseases and has been associated with altered serum amino acid levels. However, the direct role of the gut microbiome in these changes remains unconfirmed. This study employs genome-scale metabolic modeling to predict amino acid auxotrophy and peptidase gene profiles in the small intestinal microbiomes of SIBO and non-SIBO subjects. Auxotrophy and peptidase gene profiles were further examined in the large intestinal microbiome under non-dysbiotic conditions to assess their similarity to the microbial SIBO profile. Our analysis revealed that the abundance of auxotrophic bacteria is higher in the microbiota of the small intestine than in the large intestine in non-dysbiotic controls. In patients with SIBO, the abundance of auxotrophies in the small intestine decreased compared to non-SIBO subjects. Peptidase gene profiles in non-dysbiotic individuals were distinct between small and large intestinal microbiomes, with fewer extracellular peptidase genes in small intestine microbiomes. In SIBO, extracellular peptidase genes increased compared to non-SIBO subjects. Further, there were more significant associations between the abundance of auxotrophies and peptidase genes in microbiomes of the small intestine compared to the large intestine. In conclusion, the auxotrophy and peptidase gene profiles of the small and large intestinal microbiomes were distinct. In SIBO, the small intestinal microbiome shifts towards a metabolic state resembling that of the large intestine, particularly in its increased abundance of extracellular peptidase genes. This highlights the potential of genome-scale metabolic modeling in identifying metabolic disruptions associated with SIBO, which could inform the development of targeted interventions.

## Graphical Abstract

**Figure:**
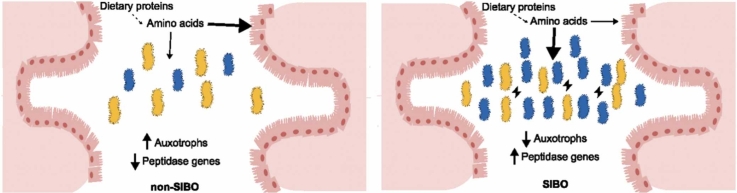

## 1 Introduction

The microbiota colonizes the entire intestinal tract, but its composition and metabolism are distinct between the small and large intestines [1]. This is driven in part by greater availability of simple nutrients, including mono- and disaccharides, free amino acids, and dietary proteins and peptides between the small and large intestines [2]. Many studies have demonstrated the involvement of gut microbial metabolism in human health [3], [4], but the focus of research tends to be on the large intestinal microbiome. As a result, the small intestinal microbiome is sometimes referred to as the "forgotten microbiome," [5] despite its well-documented role in the metabolic transformations of dietary molecules and its involvement in gastrointestinal disorders [6]. Studies in recent years have shown a link between the small intestinal microbiota and human health, demonstrating the importance of the small intestinal microbiota [7], [8].

Digestion of dietary nutrients results in an altered nutritional environment in the small intestine compared to the large intestine. In the small intestine, dietary proteins are hydrolyzed into smaller peptides and amino acids, which are mainly absorbed via the mucosa [9]. Given that substantial amount of degradation and uptake, the microbiota residing in the lower intestinal tract has limited access to proteins, peptides, and amino acids in contrast to the microbiota of the small intestine [10], [11], [12]. As a result, proteins and peptides of both human and bacterial origin are crucial sources of amino acids in the lower gastrointestinal tract [11], [13], [14]. In fact, bacteria in the large intestine secrete more proteins than those of the small intestine [15], which may reflect increased interdependence of microbes within the large intestinal milieu.

Dietary proteins and peptides are hydrolyzed not only by human proteases but also by proteases and peptidases produced by the human gut microbiome [16]. Proteolysis releases amino acids and peptides, which are essential nutrients for many gut-dwelling microorganisms [17]. The decreasing availability of free amino acids along the gastrointestinal tract highlights the importance of proteolytic activity for microbial amino acid acquisition [12], [18]. The reduction in free amino acids will have outsized effects on those bacteria that are auxotrophic for particular amino acids; e.g., for lysine auxotrophs, which by definition cannot produce lysine *de novo* and are therefore reliant on lysine from their environment. In contrast, a lysine prototroph can synthesize lysine *de novo* [17]. Theoretical and *in-vitro* studies have highlighted the importance of auxotrophies for microbial community composition [19], [20] and communal resilience to resource fluctuations [21], [22].

Since determining amino acid auxotrophies experimentally is laborious and limited to microorganisms that can be cultivated in isolation under laboratory conditions [23], computational tools were developed that aim to predict auxotrophies from genome sequences [24], [25], [26], [27]. These computational tools either use sequence homology-based approaches that predict the presence of known amino acid biosynthetic pathways [25] or employ genome-scale metabolic modeling to predict growth phenotypes under different growth media conditions [24], [27]. The application of metabolic modeling-based auxotrophy prediction tools has indicated that amino acid auxotrophies are prevalent among human gut bacteria, that the auxotrophy predictions have high accuracy when compared to experimentally determined auxotrophies, and that amino acid auxotrophies are positively associated with gut microbiome diversity and long-term stability [24]. However, these studies focused on the large intestinal microbiome. The auxotrophy profile of the small intestinal microbiome might differ due to the higher availability of amino acids compared to the colon ecosystem. Moreover, microbial proteolytic activity might be important for the release of free amino acids from peptides of non-dietary origin for auxotrophs in the small intestine. While proteolytic activity has been experimentally studied in lactic acid bacteria in the context of amino acid auxotrophies [28], [29], its role within the microbial ecosystems of the human small intestine remains largely unexplored.

Many diseases are characterized by altered amino acid metabolism of the small intestinal microbiota, for example, hyperglycemia and obesity [30], [31]. In liver cirrhosis patients, next to an alteration of the amino acid metabolism, there is also an enrichment in peptidases in the duodenal microbiota [32]. Microbial proteolytic activity may impact host-microbiome interactions in certain diseases. For instance, small intestinal bacterial overgrowth (SIBO) is a dysbiosis of the small intestinal microbiota characterized by an abnormal density of bacteria ≥ 10^5^ CFU/ml [33]. SIBO is associated with several diseases, including nonalcoholic fatty liver disease [34] and obesity [35], and is often accompanied by protein malabsorption [33], [36]. A recent study showed that alterations in the levels of tryptophan and its derived metabolites are associated with mental health conditions in patients with SIBO [37]. Symptoms were reduced after antibiotic treatment targeting the small intestinal microbiota, suggesting that the metabolic activity of bacteria in the small intestine contributes to symptoms associated with SIBO. However, whether there is an imbalance in the small intestinal microbiota's protein and amino acid metabolism in patients with SIBO remains unclear.

**Fig. 1:**
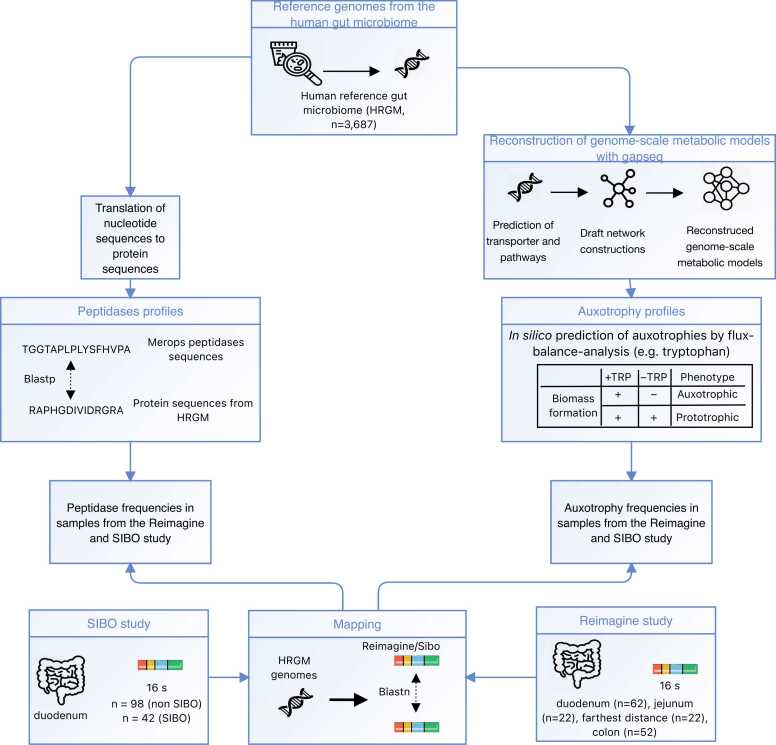
Flowchart of the study. Genome-scale metabolic models were reconstructed from genomes of the human reference gut microbiome [38] (HRGM) catalog with gapseq [39]. Auxotrophies were predicted with metabolic modeling using flux-balance analysis. The peptidase profiles were determined by scanning the peptidase sequences of the HRGM catalog for peptidases from the MEROPS database [40]. The distribution of peptidases and auxotrophies was predicted in the SIBO and Reimagine study cohorts after mapping on HRGM genomes. The farthest distance refers to the distance that could be reached during the sampling. Free available icons were taken from www.flaticon.com (creators: photo3idea_studio, surang, Icon home Eucalyp, Kiranshastry, Becris, Nadiinko).

This study builds on our previous work [24], which investigated auxotrophies in the large intestinal microbiome under non-dysbiotic conditions. Here, we build on these findings by investigating the influence of varying nutritional conditions along the gastrointestinal tract on the distribution of auxotrophies by analyzing publicly available microbiome data obtained from small intestine regions (i.e., duodenum, jejunum, and ileum) and the large intestine not only in both dysbiotic (i.e., SIBO) and non-dysbiotic states. Auxotrophies were predicted with genome-scale metabolic modeling (Fig. 1). In addition, we compared the predicted peptidase gene profiles in microbiomes of the duodenum, jejunum, ileum, and the large intestine by scanning for peptidases based on the MEROPS proteolytic enzyme database and assessed the potential relationship between auxotrophy abundances and peptidases. Furthermore, we investigated the peptidase and auxotrophy profiles of the microbiome in SIBO.

## 2 Results

### 2.1 Cohorts and microbial genome quantification

For this study, publicly available microbiome sequencing data of two cohorts (“Reimagine study” [41] and “SIBO study” [42]) were re-analyzed. The “Reimagine study” collected microbiome samples from the stool, duodenum, jejunum, and farthest distance measured during sampling (farthest distance). The “SIBO study” took samples from the duodenum of patients with SIBO and a non-SIBO control group [42]. For our re-analysis, amplicon sequence variants (ASVs) were inferred from the 16S rRNA gene sequencing data. Representative sequences of ASVs were mapped to reference genomes of the Human Reference Gut Microbiome (HRGM) catalog [38]. The amino acid auxotrophy profiles were predicted by genome-scale metabolic modeling, as described recently [24]. The inferred relative abundances of individual genomes from the HRGM catalog per sample can be found in Supplementary Tables S1 and S2.

### 2.2 Auxotrophies are more abundant in the small intestinal microbiome than in the large intestinal microbiome

**Fig. 2:**
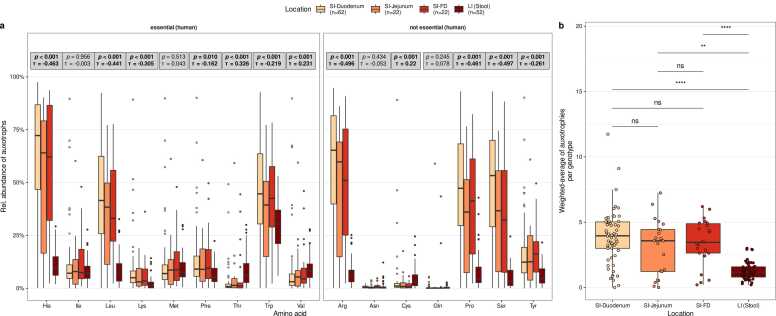
Comparison of auxotrophy profiles in small and large intestinal microbiomes predicted by genome-scale metabolic modeling. (a) Comparison of individual microbial amino acid auxotrophy abundances in the SI (Small intestine)-duodenum, SI-jejunum, SI-FD (farthest distance reached in the small intestine), and large intestine (LI). Grey boxes report p values and Kendall’s τ (tau) from Kendall rank sum correlation tests between the relative abundance of auxotrophies (y-axis) and the intestinal regions (color-coded), which is treated as an ordered factor. Reported p values are FDR-adjusted. (b) Abundance-weighted average of auxotrophies per genotype in the duodenal, jejunal, farthest distance, and colonic microbiomes. Horizontal bars denote pairwise tests for statistical differences using Wilcoxon rank sum tests. Labels above bars indicate significance levels: ns – not significant (p value > 0.05), * * (p < 0.01), * ** * (p < 0.0001).

Auxotrophy abundances were compared between gastrointestinal locations: duodenum, jejunum, farthest distance, and large intestine (Fig. 2). Here, *auxotrophy abundance* refers to the summed relative abundance of genotypes (representative HRGM genomes) auxotrophic for a specific amino acid in a given microbiome sample. Overall, the results show that microbiomes in the small intestine have higher auxotrophy abundances than microbiomes in the large intestine (Fig. 2b). The abundance-weighted average of the number of auxotrophies per genotype is higher in the microbiomes of all three small intestinal locations (duodenum, jejunum, farthest distance) compared to large intestinal microbiomes (Wilcoxon rank sum test, FDR-corrected p values < 0.05). The "*abundance-weighted average of the number of auxotrophies per genotype*" is defined as the weighted mean of the number of amino acid auxotrophies (0–20) across all bacterial species in a microbiome sample, where species’ relative abundances serve as weights to reflect their contribution to the overall auxotrophy profile. 9 out of the 16 tested amino acid auxotrophies significantly decreased in their relative abundances along the intestinal tract, with higher levels in the small intestine and lower relative abundance in the large intestine (Kendall’s rank correlation test with intestinal locations treated as ordered factors, τ < 0, *p*_adj_ < 0.05, Fig. 2a). These amino acids included the human essential amino acids His, Leu, Lys, Phe, and Trp and the non-essential amino acids Arg, Pro, Ser, and Tyr. In contrast, three amino acid auxotrophies increased in relative abundance along the intestinal tract, with the highest levels in the large intestine: Thr, Val, and Cys (Kendall’s rank correlation test, τ > 0, *p*_adj_ < 0.05, Fig. 2a). No statistically significant differences were observed for the abundance-weighted average of auxotrophies between the microbiomes of the different small intestinal regions (duodenum, jejunum, farthest distance, Fig, 2b).

### 2.3 Distinct peptidase gene profiles of the small and large intestinal microbiomes

**Fig. 3:**
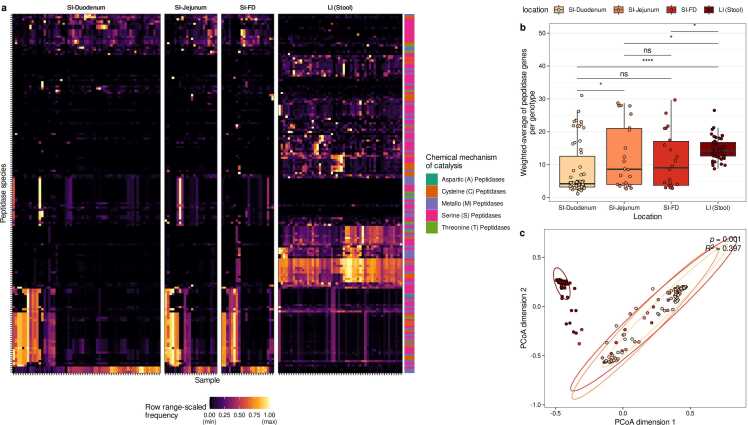
Comparison of peptidase gene profiles in small and large intestinal microbiomes. (a) Heatmap of individual peptidase abundances (range-scaled) in the microbiomes of the small intestine(SI)-duodenum, SI-jejunum, SI-farthest distance (SI-FD), and large intestine (LI) microbiomes. (b) Abundance-weighted average of peptidases. Horizontal bars denote pairwise tests for statistical differences using Wilcoxon rank sum tests. Labels above bars indicate significance levels: ns – not significant (p value > 0.05), * (p < 0.05), * ** * (p < 0.0001). (c) PCoA plot of peptidase profiles of the microbiomes of the four different intestinal regions (PERMANOVA based on Bray-Curtis Distances of pairwise peptidase profiles, R^2^=0.397, p = 0.001).

Next, we compared the distribution of genes encoding putative extracellular peptidases within the microbiome inhabiting various gastrointestinal regions (duodenum, jejunum, farthest distance, large intestine; Fig. 3, Supplementary Table S3, S4). Overall, the distribution of peptidases was clearly distinct between the large and small intestinal microbiomes (Fig. 3a). Among all peptidases with a median relative abundance of at least 1 % in either the large intestine, duodenum, or both, 76 significantly differed in abundance between the large intestine and duodenum (p < 0.001, Wilcoxon signed-rank test; Supplementary Table S4). Here, the term ‘*relative peptidase abundance’* is defined as the summed relative abundance of genotypes (i.e., representative HRGM genomes) that harbor at least one homologous gene for a given peptidase species. The most prevalent peptidase family among the 76 differentially abundant peptidases was the S09 (prolyl oligopeptidase) family, which had 12 hits. All S09 hits were significantly more abundant in the large intestine than in the duodenum group (Supplementary Table S4). The differences between gene profiles of extracellular peptidases were also evident in a Principal Coordinate Analysis (PCoA) using the Bray-Curtis Distance as metric to quantify distances between gene profiles (PERMANOVA, R^2^ = 0.397, p = 0.001, Fig. 3c). Moreover, the genotype abundance-weighted averages of the number of genes encoding extracellular peptidases indicated that these peptidase genes are less common in microbiomes from each small intestine region compared to the large intestine microbiome (Wilcoxon rank sum test, p < 0.05; Fig. 3b). When comparing extracellular peptidase gene profiles between microbiomes of the small intestine, we observed that the microbiomes in the jejunum harbor more peptidase genes than in the duodenum (p = 0.02985, Fig. 3b). In sum, the results indicate that the peptidase gene profile of the small intestine microbiome is distinct from that of the large intestine, and the large intestinal microbiomes harbor more genes encoding putative extracellular peptidases.

### 2.4 Abundances of auxotrophies are often negatively associated with abundances of peptidase genes in the microbiomes of the small and large intestine

**Fig. 4:**
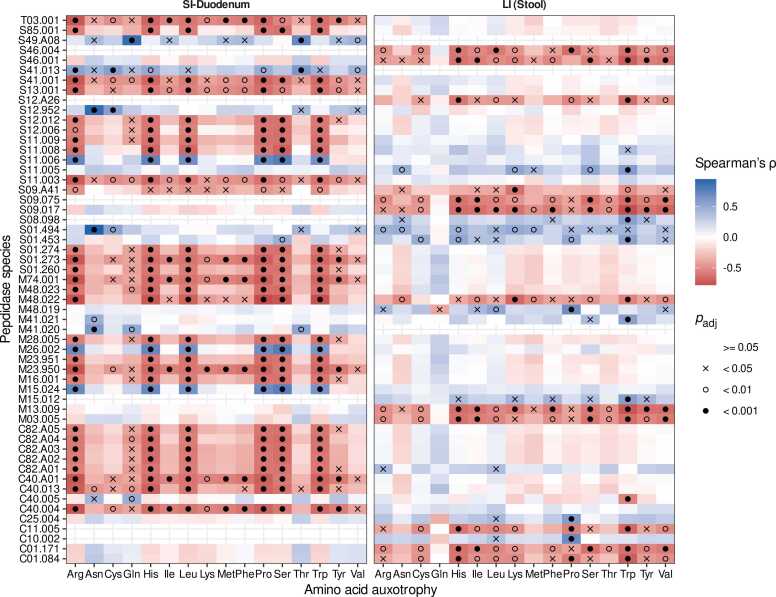
Associations between predicted peptidases and auxotrophies in duodenal (SI-Duodenum) and large intestinal (LI-stool) microbiomes. Spearman rank sum correlations were calculated between the relative abundance of the respective amino acid auxotrophy (x-axis) and the relative abundance of specific peptidases (y-axis). p values were FDR-adjusted.

Hydrolysis of extracellularly accessible peptides could be a mechanism for auxotrophic bacteria to cover their demand for amino acids. Therefore, we tested for the potential association between the relative abundance of individual amino acid auxotrophies and the relative abundance of the identified putative extracellular (Fig. 4). The correlation analysis was performed separately for large intestine microbiome samples and duodenal microbiome samples because the duodenum was the small intestinal region with the largest sample size. More statistically significant correlations between auxotrophies and peptidase genes were found in the duodenum than in the large intestine (Spearman’s rank correlation, Fig. 4). Additionally, most correlations were negative between abundances of auxotrophies and peptidase genes. Auxotrophies for arginine, histidine, leucine, proline, serine, and tryptophan had the most statistically significant correlations with peptidase genes in the duodenum. In the duodenum, several auxotrophies were negatively associated with peptidases involved in antibiotic resistance (S11, S12, S13 peptidase families), cell wall synthesis (S11, S12, C82, C40 peptidase families), and peptidoglycan synthesis (C82 peptidase families). Further, auxotrophies were negatively correlated with peptidase genes involved in glutathione degradation (T03.001), dietary protein degradation (S01.260), insulin and beta-amyloid degradation (M16.001), and the lysis of cell walls (M23.950, M23.951). In large intestinal microbiomes, peptidases involved in the degradation of oligopeptides degradation (S09.075, S09.A41, S09.017) and anti-microbial peptides (C01.171) were negatively associated with several amino acid auxotrophies (Fig. 4).

In conclusion, auxotrophies are mostly negatively correlated with peptidase genes in small and large intestinal microbiomes.

### 2.5 Auxotrophies are reduced, while extracellular peptidase gene abundances are increased in the small intestinal microbiomes of SIBO patients

SIBO is a dysbiotic state of the small intestine and is associated with several gastrointestinal disorders. Serum and urine levels of tryptophan and its derivatives were altered in SIBO patients, but direct involvement of the gut microbial amino acid metabolism was not studied [37]. Therefore, we reanalyzed duodenal samples from a SIBO study [42] and compared auxotrophy and peptidase gene profiles in the duodenum of SIBO and non-SIBO subjects.

**Fig. 5:**
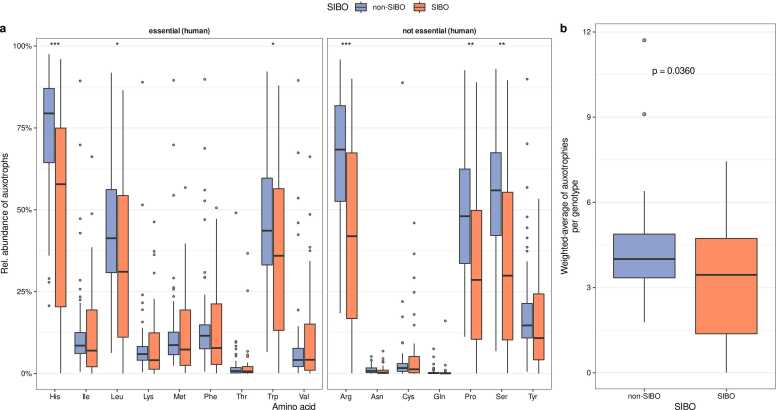
Genome-scale metabolic modeling predicted auxotrophy profiles in the duodenal microbiomes of non-SIBO and SIBO samples*.* (a) Individual amino acid auxotrophy abundances in the duodenal microbiome of SIBO and non-SIBO patients. Asterisks denote the statistical significance of differences in the relative abundance of the respective auxotrophy between the SIBO and the non-SIBO group (Wilcoxon rank sum test, * p < 0.05, ** p < 0.01, *** p < 0.001). p values are FDR-adjusted. (b) Abundance-weighted average of the number of auxotrophies per gut microbial genotype in SIBO and non-SIBO (Wilcoxon rank sum test, p = 0.036).

So far, we have observed that the auxotrophy abundances in the non-dysbiotic duodenal microbiome are higher than in the large intestinal microbiome (Fig. 2a). In the dysbiotic state of the duodenal microbiome of SIBO subjects, we observed that auxotrophy abundances are decreased compared to non-SIBO subjects. Auxotrophy abundances of the essential amino acid histidine, leucine, and tryptophan, as well as for the non-essential amino acids proline, arginine, and serine, were lower in the microbiomes of SIBO subjects compared to non-SIBO subjects (Fig. 5a, Wilcoxon rank sum test, FDR corrected p value < 0.05). The abundance-weighted average of auxotrophies was decreased in the microbiome of SIBO patients (Fig. 5b, Wilcoxon rank sum test, p = 0.0360).

**Fig. 6:**
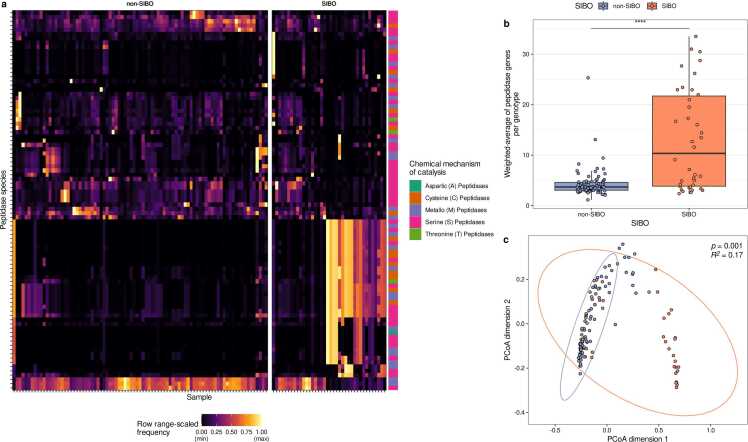
Microbial peptidase gene profiles in duodenal microbiomes of SIBO and non-SIBO samples. (a) Heatmap of individual peptidase abundances (range-scaled) in small intestinal microbiomes from SIBO and non-SIBO samples. (b) Abundance-weighted average of peptidases in the microbiomes of SIBO and non-SIBO samples (Wilcoxon rank sum test, p value < 0.0001). (c) PCoA plot of peptidase profiles of the microbiomes of SIBO and non-SIBO samples (PERMANOVA based on Bray-Curtis Distances of pairwise peptidase profiles, R^2^=0.17, p = 0.001).

Further, the peptidase gene profiles were compared between microbiomes of non-SIBO and SIBO subjects. Some peptidases were exclusively detected in either the SIBO or non-SIBO microbiomes (Fig. 6a, Supplementary Table S5, S6). PCoA indicated that the peptidase profiles are distinct between the microbiomes of non-SIBO and SIBO subjects (Fig. 6c, PERMANOVA, R^2^ = 0.17, p = 0.001). Further, the abundance-weighted average of putative extracellular peptidases was higher in the microbiomes of SIBO patients compared to non-SIBO samples (Wilcoxon rank sum test, p = 0.028, Fig. 6b). In brief, auxotrophy abundances are reduced, but extracellular peptidase genes are more abundant in the microbiomes of SIBO patients.

### 2.6 Small intestine microbiomes in SIBO patients show slightly greater similarity to large intestine microbiomes in auxotrophy and peptidase gene profiles compared to non-SIBO individuals

**Fig. 7:**
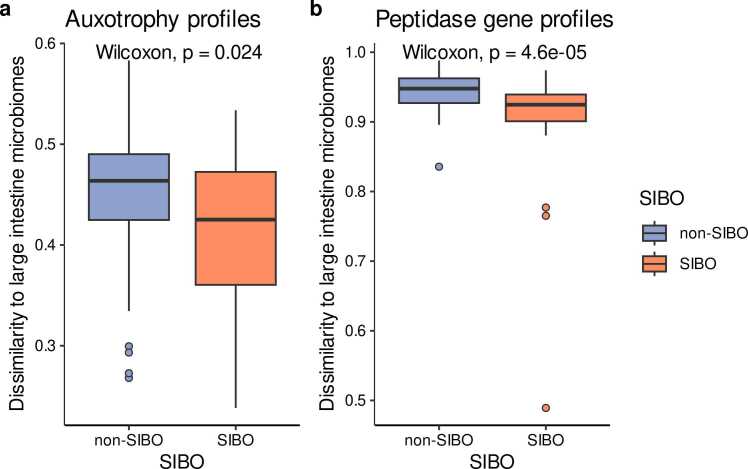
Dissimilarities of amino acid auxotrophy (a) and peptidase gene profiles (b) of small intestine microbiomes to profiles of large intestine microbiomes. Small intestine microbiomes in SIBO patients show slightly higher similarity to large intestine microbiomes in auxotrophy and peptidase gene profiles than non-SIBO individuals. The y-axis displays the median Bray-Curtis-Dissimilarities for each small intestine microbiome sample from the patients with SIBO to all large intestine microbiomes from non-dysbiotic individuals. The SIBO and non-SIBO groups were compared by Wilcoxon rank sum tests.

Bacterial imbalances observed in SIBO are partly a result of translocation of colonic bacteria to the small intestine [43]. Here, we wanted to test whether this is also reflected in amino acid auxotrophy and peptidase gene profiles when comparing small intestinal microbiomes from SIBO and non-SIBO subjects to the microbiomes from the large intestine. To this end, we calculated the median Bray-Curtis-Dissimilarity scores between the auxotrophy and peptidase gene profiles of the small intestine samples from the SIBO study and large intestine microbiome samples from the Reimage study (Fig. 7). Amino acid auxotrophy profiles in individuals with SIBO showed significantly greater similarity to those found in large intestinal microbiomes when compared to non-SIBO samples (Wilcoxon rank sum test, p = 0.024; Fig. 7a). This resemblance is also reflected in the lower relative abundances of auxotrophies for histidine, leucine, tryptophan, arginine, proline, and serine in SIBO compared to non-SIBO (Fig. 6a). However, the reduced auxotrophy abundances in SIBO patients are still substantially higher than the relative abundances observed in the large intestinal microbiomes (Fig. 2a). Peptidase gene profiles in the small intestine from patients with SIBO also showed higher similarity to large intestine microbiomes than peptidase gene profiles from non-SIBO subjects (p = 4.6 ×10^−5^; Fig. 7b). However, with a median Bray-Curtis-Dissimilarity of > 0.9, the peptidase gene profiles in SIBO samples remain distinct from profiles of large intestine microbiomes.

## 3 Discussion

Here, we compared the auxotrophy and extracellular peptidase gene profiles of the microbiomes in the small and large intestine. We additionally investigated whether the profiles exhibited distinct patterns in the dysbiotic state of SIBO compared to non-SIBO subjects. Auxotrophy abundances were higher in the microbiomes of the small intestine than the large intestine, which aligns with the hypothesis that the small intestinal microbiome secures more amino acids from host dietary sources than the large intestinal microbiome. In the dysbiotic state SIBO, auxotrophy abundances were reduced in the duodenal microbiome. Many peptidase genes were specific to either the small or large intestinal microbiome, suggesting that the availability of different protein and peptide sources along the digestive tract shapes the microbial peptidase gene profile. The abundance-weighted average of extracellular peptidase genes was reduced in the small intestinal microbiomes than the large intestinal microbiomes but increased in the microbiomes of SIBO patients.

Our study provides a comprehensive overview of the distribution of peptidases and amino acid auxotrophies in the small and large intestine microbiome in non-dysbiotic and dysbiotic states.

### 3.1 The amino acid-rich environment in the small intestine might promote higher auxotrophy abundances in non-dysbiotic microbiomes

The higher availability of free amino acids in the small intestine resulting from the digestion of dietary proteins [10], [44] suggests that the microbiome’s dependence on extracellularly accessible amino acids is higher in the small than in the large intestine. Our results support this hypothesis, as we observed higher auxotrophic frequencies for nine amino acids (tryptophan, leucine, histidine, lysine, phenylalanine, proline, arginine, tyrosine, and serine) in microbiomes of the small intestine compared to the large intestine (Fig. 2a). This suggests that the growth conditions provided by the amino acid-rich environment in the small intestine might confer a fitness advantage for auxotrophic bacteria over prototrophic bacteria. When the essential nutrients are provided, auxotrophic bacteria tend to have a fitness advantage over prototrophic bacteria [45]. This could be particularly beneficial in situations for the large and energetically costly amino acids (e.g., tryptophan, histidine) [46]; indeed, these amino acid auxotrophies were particularly common. Additionally, tryptophan and histidine are slowly absorbed via the small intestine mucosa compared to other proteinogenic amino acids and might be available longer for uptake by the small intestine microbiome [47]. Interestingly, amino acid auxotrophies for threonine, methionine, phenylalanine, and cysteine were higher in large intestinal microbiomes. Threonine is available in the large intestine in the backbone of mucin glycoproteins. For example, the threonine auxotroph *Akkermansia muciniphila* can degrade mucin glycoproteins [14]. Cysteine and methionine are present in the colonic mucosa as intermediates of the homocysteine cycle [48], and by their secretion to the lumen, they could be utilized by cysteine and methionine auxotrophic bacteria. Cysteine is secreted from the mucosa into the small intestinal lumen [49]. In summary, the heterogeneous auxotrophy profiles of the small and large intestines are primarily attributed to their distinct microenvironments, which results in differential amino acid availability.

### 3.2 Peptidase gene profiles are distinct between small or large intestinal microbiomes

Our results indicated distinct profiles of extracellular peptidase genes in small and large intestinal microbiomes (Fig. 3). The diverse nutritional conditions along the gastrointestinal tract create distinct ecological niches, which are likely occupied by different bacterial species [50]. This ecological variation is a plausible factor contributing to the distinct peptidase profiles observed in the small and large intestinal microbiomes. Enzyme repertoire studies on human gut microbiome isolates suggest microbial specialization for protein sources in several different microbial species [51], [52]. For example, *Akkermansia muciniphila*, a known colonizer of the large intestine, harbors a repertoire of peptidases for mucin glycoprotein degradation [51]. Similarly, *Paraprevotella* species are recognized as trypsin degraders in the large intestine [52]. Various other bacterial strains are capable of gluten degradation in the duodenum [53]. Thus, bacteria in the human gut might specialize in protein sources tailored to their microenvironment, which explains the repertoire of specific peptidases along the gastrointestinal tract.

### 3.3 Abundances of auxotrophic bacteria are negatively correlated with several peptidase genes in small and large intestinal microbiomes

The microbial peptidase activity is linked to the composition of the gut microbiome [54]. It might play an important in degrading available proteins and peptides, thereby releasing amino acids that can serve as essential nutrients for auxotrophic bacteria. Thus, proteolytic activity contributes to the availability of amino acids for the intestinal microbiome, and we hypothesized that the abundance of genes encoding extracellular peptidases in the microbiome would be positively associated with the abundance of auxotrophies. Therefore, we correlated auxotrophy abundances with peptidase gene abundances in duodenal and large intestinal microbiomes. Contrary to our hypothesis, we observed predominantly negative correlations between the relative abundance of amino acid auxotrophies and peptidase genes in small and large intestinal microbiomes (Fig. 4). Auxotrophic bacteria in the small intestine might save metabolic costs by abstaining from proteolysis since they thrive in the nutrient-rich environment of the small intestine with accessible free amino acids derived by dietary protein digestion facilitated by peptidases secreted by the human host. However, this does not explain the large number of negative correlations between peptidase genes and auxotrophic bacteria in the large intestine. Amino acid cross-feeding between prototrophic and auxotrophic genotypes might be one contributing factor, as this potential source of amino acids for auxotrophic genotypes does not necessarily require proteolytic activity. In addition, prototrophic bacteria that express extracellular peptidases can turn off the expression of genes for amino acid biosynthesis when free amino acids are available from proteolysis.

Our study shows that the abundance of auxotrophies in small and large intestinal microbiomes is negatively correlated with the abundance of many peptidase genes, which indicates that auxotrophic bacteria in the gut might commonly utilize different mechanisms of amino acid acquisition than proteolysis.

### 3.4 Increased abundances of prototrophic bacteria and extracellular peptidase genes are characteristics of the microbiome of SIBO patients

The small intestinal microbiome is still understudied compared to the large intestinal microbiome [6]. Several studies indicated that the small intestinal microbiome is involved in the pathophysiology of specific diseases [7], [8], [31], [42]. SIBO is characterized by an abnormal growth (>10^5^ organisms/ml) of bacteria [55] and is associated with several diseases. However, the ecology of bacteria within the gut microbiome of patients with SIBO remains largely unknown [43], [56]. Investigating the nutritional dependencies of small intestinal bacteria through the study of amino acid auxotrophies could provide new insights into the ecology of the small intestinal microbiome in SIBO, and improve our understanding of its role in the development of SIBO.

Here, we compared the amino acid auxotrophy profiles of patients with SIBO and non-SIBO controls. The abundance-weighted average of auxotrophies was reduced in the microbiome of patients with SIBO (Fig. 5b). This is in line with previous findings demonstrating increased small intestine colonization with colonic bacteria in SIBO [57], since we also observed that bacteria of the large intestinal microbiome commonly have less amino acid auxotrophies compared to the small intestine (Fig. 2b). Moreover, the microbiomes of the small intestine in SIBO patients exhibited a greater similarity to the microbiomes of the large intestine in terms of auxotrophy and peptidase gene profiles compared to non-SIBO individuals, further suggesting that small intestine colonization with colonic bacteria (Fig. 7). There could be several potential causes for the colonization of the small intestine with atypical bacteria from the colon environment in SIBO. SIBO is frequently seen in patients with reduced gastric acid, which could lead to an increased pH in the small intestine compared to healthy individuals and creates a growth environment closer to the one found in the large intestine. Therefore, more colonic bacteria might be able to survive in this less acidic environment. Further, the motility of the gut of SIBO patients is commonly reduced [58]. This might lead to less bacterial translocation from the small intestine to the large intestine [59]. Stasis is frequently observed in patients with SIBO [60], [61], further suggesting a slower transit time of food through the gastrointestinal tract, allowing nutritional resources to remain available for bacteria in the small intestine for a longer period. This could promote the overgrowth of atypical bacteria in the small intestine, which is in a non-dysbiotic state characterized by an acidic environment with quickly changing nutritional conditions [1].

While several factors may promote the colonization of canonically colonic bacteria in the small intestinal microbiome of SIBO patients, the relationship between these factors and auxotrophies, and the reasons for the contrasting abundance of auxotrophies in non-dysbiotic versus dysbiotic small intestinal microbiomes, remain unexplored. Auxotrophic bacteria likely utilize free amino acids released during dietary protein digestion by proteolytic enzymes secreted into the gut lumen by the human host. While prototrophic bacteria can also access these amino acids, auxotrophic bacteria may have a fitness advantage in the nutrient-rich conditions [45] of the small intestine, which could explain their higher abundance there. Moreover, the overgrowth of bacteria in SIBO could lead to limited nutritional resources through increased nutrient competition between bacteria. Therefore, the gut environment in SIBO patients might favor prototrophic bacteria because they can synthesize specific growth-essential nutrients (i.e., amino acids) and are less dependent on their nutritional environment compared to auxotrophic bacteria. In sum, while free amino acids available by dietary protein digestion may support the survival of auxotrophic bacteria in the small intestine in a non-dysbiotic state, the shifts in nutrient availability and competition in SIBO favor prototrophic bacteria. Thus, increased prototrophic and reduced auxotrophic bacteria in SIBO can be explained by the changed nutritional landscape in this dysbiotic state.

Our analysis indicates that the small intestinal microbiome in patients with SIBO has a higher abundance of genes encoding potential extracellular-acting peptidases compared to non-dysbiotic controls. This increase suggests that SIBO-associated bacteria may engage in more aggressive nutrient acquisition strategies, potentially "competing" with the host for nutrients, such as by the uptake and catabolism of amino acids. Supporting this, Chojnacki et al. (2022) reported that urinary tryptophan levels increased in patients with SIBO following antibiotic treatment [37]. Therefore, the elevated abundance of extracellular peptidase genes may indicate enhanced bacterial peptidolysis, potentially leading to protein deficiency symptoms in patients with SIBO.

In summary, in a non-dysbiotic state, auxotrophic bacteria are increased in small intestinal microbiomes compared to large intestinal microbiomes, but they are decreased in a dysbiotic SIBO. The microenvironment in the microbiome of SIBO patients favors prototrophic bacteria and increased extracellular peptidase genes in bacteria.

### 3.5 Limitations

There are some limitations in our study. Predictions of auxotrophy and peptidase profiles are based on the mapping of 16S rRNA sequences to human gut reference microbiome sequences. This was necessary due to the paucity of published whole genome sequences from small intestinal microbiomes; predictions are therefore based on taxonomic information. Also, the presence of peptidase genes was analyzed in small and large intestinal microbiomes in dysbiotic and non-dysbiotic states, but the approach cannot conclude the actual peptidolytic activity.

## 4 Conclusion

The nutritional requirements in the small intestinal microbiome are still understudied despite its association with the pathophysiology of several diseases. This study showed that the small intestinal microbiomes have higher nutrient requirements, as indicated by increased amino acid auxotrophies, than the large intestinal microbiome in non-dysbiotic states. However, amino acid auxotrophies are reduced in the small intestinal microbiome in small intestinal bacterial overgrowth (SIBO), underlining different nutrient requirement patterns in dysbiotic states. Our study further suggests a disrupted microbial ecology in SIBO patients, highlighted by increased extracellular peptidase genes in their microbiome. As alternatives to antibiotics are increasingly sought for SIBO treatment, understanding the small intestinal microbiome's nutritional requirements holds great potential for developing effective therapies. Therefore, the results of this study could advance the knowledge to restore microbial balance and improve therapeutic outcomes for patients with SIBO.

## 5 Material and methods

### 5.1 Cohorts and 16S rRNA gene sequencing analysis

16S rRNA gene sequencing data were re-analyzed from two previous studies. The first study took samples from four gastrointestinal sites: the duodenum, jejunum, farthest distance, and stool [41]. The farthest distance refers to the location in the gastrointestinal tract that could be reached during sampling. The second study investigated the duodenal microbiome in SIBO [42]. Information about the sampling method is provided by the authors of the studies [41], [42]. Both studies were conducted in the United States of America at the Cedars-Sinai Medical Center. In both studies, 16S rRNA gene sequencing was used for microbiome profiling.

The 16S rRNA gene sequencing data from the first study were downloaded with the SRA toolkit [62] (version 3.0.0) from NCBI (BioProject ID: PRJNA590519) [41]. 16S rRNA gene sequencing data from the SIBO study were taken from the ENA Server (BioProject ID: PRJNA525828) [42]. Primer sequences were detected and removed from read sequences using the software Cutadapt [63]. Sequences were only kept for further analysis if the forward and reversed primer were found and removed. After trimming, the sequence length was shortened to 275. The package DADA2 (version 1.24.0) was used to generate the ASVs [64]. After checking the quality of the reads, forward reads with a length of 265 and reverse reads with a length of 245 were kept (Tolerated maximal errors (maxEE = 2) from the first study of the sample taken from four gastrointestinal sites. For the SIBO study, the forward reads were filtered for the length of 240 and reverse reads for 230 (maxEE = 2).

### 5.2 Mapping to representative genomes of the human gut microbiome

Representative nucleotide sequences of the calculated ASVs were mapped to the 16S rRNA genes within genomes of the Human Reference Gut Microbiome (HRGM) catalog, which consists of 5414 distinct prokaryotic species [38], [65]. The mapping was based on pairwise sequence alignments using BLASTN (version 2.9.0 +, [66]) with a minimum query (ASV) coverage of 95 % and a minimum sequence identity of 97 %. In the case of multiple hits, only hits with the maximum identity were retained for further analysis.

### 5.3 Prediction of peptidases

The MEROPS peptidase unit database harbors information about peptidase units [40]. All protein coding genes and their translation to amino acid sequences within the genomes of the HRGM collections were predicted using PRODIGAL (version 2.6.3) [67]. For the prediction of peptidases in the HRGM sequences, a BLASTp search was performed with the MEROPS scan sequences (e-value < 0.01, query coverage > 85 %) [68]. After the blast search, only hits with a bitscore higher than 100 and a coverage of the MEROPS peptidase unit of 95 % or higher were considered as putative peptidases in the HRGM genomes. Next, we wanted to identify those peptidase genes in the HRGM genomes, that likely encode peptidases that act on extracellular peptides (e.g., via enzyme secretion or cell surface-attached enzymes). Therefore, we used SignalP (v6.0) [69] to predict signal peptides in the peptidase amino acid sequences that indicate enzymes that are putatively secreted or translocated across the cytoplasmic membrane. All signal peptides included in SignalP 6.0 were included in the analysis. Peptidase abundances refers to the relative abundance of each peptidase in a sample. The abundance-weighted average of peptidases in a sample is calculated with the equation for the abundance-weighted average of auxotrophies and can be found in our previous work [24].

### 5.4 Reconstruction of genome-scale metabolic models

Genome-scale metabolic models of individual genomes from the HRGM collection were reconstructed using gapseq (version 1.2, commit 13d88a68) [39]. All metabolic models as well as the exact gapseq commands for the reconstructions are available at Zenodo [70]. In total, 5414 genome-scale metabolic models were build but since only genomes with a completeness > =85 % were considered for the auxotrophy analysis, only 3687 models were used for the analysis. Models were reconstructed following the following steps: 1) Prediction of pathways and transporters, 2) Construction of a draft metabolic network, 3) genome-guided growth medium prediction, 4) Gap-filling. More information about the reconstruction process of the genome-scale metabolic models can be found in our previous work [24], which has used the same models that we used for the present study. There, also the growth medium prediction is descried in detail, which is crucial for growth phenotype and auxotrophy prediction using genome-scale metabolic models.

### 5.5 Prediction of auxotrophies

Auxotrophies were predicted in R (4.3.0) with the R package sybil [71] (2.2.1) using metabolic modeling as described previously [24]. Flux balance analysis was performed with the objective function defined as the flux through the biomass reaction [72]. The growth of the models was tested with and without the focal amino acid in the *in silico* growth medium. The growth medium was predicted by gapseq [39]. When no growth of the model without the focal amino acid in the medium was determined compared to the growth rate with the medium containing the amino acids, it was classified as auxotrophic for the amino acid tested.

### 5.6 Statistical analysis

All statistical analysis was performed with R (4.3.0). The correlation of auxotrophy abundances with the gastrointestinal region (ordered factors: 1 - duodenum, 2 - jejunum, 3 - farthest distance reached in the small intestine, and 4 - large intestine) was determined with the Kendal Rank Correlation, and *p* values were adjusted for multiple testing using the false discovery rate (FDR) method by Benjamini and Hochberg [73]. Differences in auxotrophy abundances of the microbiome in SIBO and non-SIBO were evaluated with the Wilcoxon rank sum test, and p values were FDR-adjusted. Differences in the abundance-weighted average of auxotrophies and peptidases in small and large intestinal microbiomes in a non-dysbiotic and dysbiotic state were determined with the Wilcoxon rank sum test. The equation for the calculation of the abundance-weighted average can be found in our previous work [24]. Differences in the abundance of peptidase in the duodenum and large intestine were compared with the Wilcoxon signed rank test, and p values were adjusted using the FDR method. An FDR-adjusted p value of < 0.05 was considered significant in all tests.

Peptidase profiles between small and large intestine microbiomes and microbiomes of SIBO and non-SIBO subjects were compared with PCoA analysis using the Bray-Curtis distance matrix. A permutational multivariate analysis of variances (PERMANOVA) as implemented in the R-package ‘vegan’ (v2.6–4) [74] was used to compare the peptidase profiles between SIBO and non-SIBO samples. The pairwise Bray-Curtis distance between peptidase profiles was used as a metric for the distance matrix.

Associations between auxotrophies and peptidases were tested using Spearman’s rank correlation.

## Code availability

The R code with information about the statistical data analysis and visualization is uploaded on GitHub: https://github.com/SvStarke/Auxos_SI_LI_microbiomes

## Data availability

16S rRNA amplicon sequencing data of the Reimagine study (BioProject ID: PRJNA590519) [41] and SIBO study (BioProject ID: PRJNA525828) [42] are available on NCBI as the data was submitted by the authors of the respective studies. Genome-scale metabolic models of the HRGM catalogue are available via Zenodo [70]. Auxotrophy predictions for all HRGM genomes can be found in the supplemental material of the published article by Starke et al., 2023 [24].

## CRediT authorship contribution statement

**Paulay Amandine:** Writing – review & editing, Methodology, Data curation. **Aden Konrad:** Writing – review & editing, Supervision, Funding acquisition. **Starke Svenja:** Writing – original draft, Visualization, Investigation, Formal analysis, Data curation, Conceptualization. **Harris Danielle MM:** Writing – review & editing, Supervision, Methodology, Conceptualization. **Waschina Silvio:** Writing – review & editing, Visualization, Validation, Supervision, Software, Resources, Methodology, Funding acquisition.

## Declaration of Generative AI and AI-assisted technologies in the writing process

During the preparation of this work, the authors used Grammarly in order to improve language and readability. After using this tool/service, the authors reviewed and edited the content as needed and take full responsibility for the content of the publication.

## Declaration of Competing Interest

The authors declare that they have no known competing financial interests or personal relationships that could have appeared to influence the work reported in this paper.

## References

[1] Seekatz A.M. (2019). Spatial and temporal analysis of the stomach and small-intestinal microbiota in fasted healthy humans. mSphere.

[2] Yersin S., Vonaesch P. (2024). Small intestinal microbiota: from taxonomic composition to metabolism. Trends Microbiol.

[3] Fan Y., Pedersen O. (2021). Gut microbiota in human metabolic health and disease. Nat Rev Microbiol.

[4] Le Chatelier E. (2013). Richness of human gut microbiome correlates with metabolic markers. Nature.

[5] Shanahan E.R., Holtmann G., Morrison M. (2017). Life in the small intestine: the forgotten microbiome?. Microbiol Aust.

[6] Chang E.B., Martinez-Guryn K. (2019). Small intestinal microbiota: the neglected stepchild needed for fat digestion and absorption. Gut Microbes.

[7] Angelakis E. (2015). A metagenomic investigation of the duodenal microbiota reveals links with obesity. PLoS ONE.

[8] Martinez-Guryn K. (2018). Small intestine microbiota regulate host digestive and absorptive adaptive responses to dietary lipids. Cell Host Microbe.

[9] Trommelen J., Tomé D., van Loon L.J.C. (2021). Gut amino acid absorption in humans: concepts and relevance for postprandial metabolism. Clin Nutr Open Sci.

[10] Chung Y.C. (1979). Protein digestion and absorption in human small intestine. Gastroenterology.

[11] Miner-Williams W. (2012). Endogenous proteins in terminal ileal digesta of adult subjects fed a casein-based diet. Am J Clin Nutr.

[12] Zeng X. (2022). Gut bacterial nutrient preferences quantified in vivo. Cell.

[13] Macfarlane G.T., Cummings J.H., Macfarlane S., Gibson G.R. (1989). Influence of retention time on degradation of pancreatic enzymes by human colonic bacteria grown in a 3-stage continuous culture system. J Appl Bacteriol.

[14] van der Ark K.C.H. (2018). Model-driven design of a minimal medium for *Akkermansia muciniphila* confirms mucus adaptation. Microb Biotechnol.

[15] Velez-Cortes F., Wang H. (2022). Characterization and spatial mapping of the human gut metasecretome. mSystems.

[16] Macfarlane G.T., Allison C., Gibson S.A.W., Cummings J.H. (1988). Contribution of the microflora to proteolysis in the human large intestine. J Appl Bacteriol.

[17] Zengler K., Zaramela L.S. (2018). The social network of microorganisms — how auxotrophies shape complex communities. Nat Rev Microbiol.

[18] Ahlman B., Leijonmarck C.-E., Lind C., Vinnars E., Wernerman J. (1993). Free amino acids in biopsy specimens from the human colonic mucosa. J Surg Res.

[19] Giri S. (2021). Metabolic dissimilarity determines the establishment of cross-feeding interactions in bacteria. Curr Biol.

[20] Oña L. (2021). Obligate cross-feeding expands the metabolic niche of bacteria. Nat Ecol Evol.

[21] Oña L., Kost C. (2022). Cooperation increases robustness to ecological disturbance in microbial cross-feeding networks. Ecol Lett.

[22] Wang, T., George, A.B.Maslov, S. Higher-order interactions in auxotroph communities enhance their resilience to resource fluctuations. Available from: 2024.05.22.595348 Preprint at https://doi.org/10.1101/2024.05.22.595348 (2024). 10.1016/j.cels.2025.101491 41720095

[23] Soto-Martin E.C. (2020). Vitamin biosynthesis by human gut butyrate-producing bacteria and cross-feeding in synthetic microbial communities. mBio.

[24] Starke S. (2023). Amino acid auxotrophies in human gut bacteria are linked to higher microbiome diversity and long-term stability. ISME J.

[25] Price M.N., Deutschbauer A.M., Arkin A.P. (2020). GapMind: automated annotation of amino acid biosynthesis. mSystems.

[26] Seif Y. (2020). Metabolic and genetic basis for auxotrophies in Gram-negative species. Proc Natl Acad Sci USA.

[27] Machado D. (2021). Polarization of microbial communities between competitive and cooperative metabolism. Nat Ecol Evol.

[28] Canon F., Maillard M.-B., Henry G., Thierry A., Gagnaire V. (2021). Positive interactions between lactic acid bacteria promoted by nitrogen-based nutritional dependencies. Appl Environ Microbiol.

[29] Tuler T.R., Callanan M.J., Klaenhammer T.R. (2002). Overexpression of peptidases in lactococcus and evaluation of their release from leaky cells. J Dairy Sci.

[30] Granata I. (2020). Duodenal metatranscriptomics to define human and microbial functional alterations associated with severe obesity: a pilot study. Microorganisms.

[31] Darra A. (2023). Hyperglycemia is associated with duodenal dysbiosis and altered duodenal microenvironment. Sci Rep.

[32] Chen Y. (2016). Dysbiosis of small intestinal microbiota in liver cirrhosis and its association with etiology. Sci Rep.

[33] Bures J. (2010). Small intestinal bacterial overgrowth syndrome. WJG.

[34] Miele L. (2009). Increased intestinal permeability and tight junction alterations in nonalcoholic fatty liver disease. Hepatology.

[35] Sabaté J.-M. (2008). High Prevalence of small intestinal bacterial overgrowth in patients with morbid obesity: a contributor to severe hepatic steatosis. Obes Surg.

[36] Klaus J. (2009). Small intestinal bacterial overgrowth mimicking acute flare as a pitfall in patients with Crohn’s Disease. BMC Gastroenterol.

[37] Chojnacki C. (2022). Antimicrobial treatment improves tryptophan metabolism and mood of patients with small intestinal bacterial overgrowth. Nutr Metab.

[38] Kim C.Y. (2021). Human reference gut microbiome catalog including newly assembled genomes from under-represented Asian metagenomes. Genome Med.

[39] Zimmermann J., Kaleta C., Waschina S. (2021). gapseq: informed prediction of bacterial metabolic pathways and reconstruction of accurate metabolic models. Genome Biol.

[40] Rawlings N.D. (2018). The MEROPS database of proteolytic enzymes, their substrates and inhibitors in 2017 and a comparison with peptidases in the PANTHER database. Nucleic Acids Res.

[41] Leite G.G.S. (2020). Mapping the segmental microbiomes in the human small bowel in comparison with stool: a REIMAGINE study. Dig Dis Sci.

[42] Leite G. (2020). The duodenal microbiome is altered in small intestinal bacterial overgrowth. PLoS One.

[43] Rao S.S.C., Bhagatwala J. (2019). Small intestinal bacterial overgrowth: clinical features and therapeutic management. Clin Transl Gastroenterol.

[44] Borgstrom B., Dahlqvist A., Lundh G., Sjovall J. (1957). Studies of intestinal digestion and absorption in the human. J Clin Invest.

[45] D’Souza G. (2014). Less is more: selective advantages can explain the prevalent loss of biosynthetic genes in bacteria: adaptive loss of biosynthetic genes in bacteria. Evolution.

[46] Waschina S., D’Souza G., Kost C., Kaleta C. (2016). Metabolic network architecture and carbon source determine metabolite production costs. FEBS J.

[47] Adibi S.A., Gray S.J., Menden E. (1967). The kinetics of amino acid absorption and alteration of plasma composition of free amino acids after intestinal perfusion of amino acid mixtures. Am J Clin Nutr.

[48] Morgenstern I., Raijmakers M.T.M., Peters W.H.M., Hoensch H., Kirch W. (2003). Homocysteine, cysteine, and glutathione in human colonic mucosa: elevated levels of homocysteine in patients with inflammatory bowel disease. Dig Dis Sci.

[49] Dahm L.J., Jones D.P. (1994). Secretion of cysteine and glutathione from mucosa to lumen in rat small intestine. Am J Physiol-Gastrointest Liver Physiol.

[50] Pereira F.C., Berry D. (2017). Microbial nutrient niches in the gut: microbial nutrient niches in the gut. Environ Microbiol.

[51] Trastoy B., Naegeli A., Anso I., Sjögren J., Guerin M.E. (2020). Structural basis of mammalian mucin processing by the human gut O-glycopeptidase OgpA from Akkermansia muciniphila. Nat Commun.

[52] Li Y. (2022). Identification of trypsin-degrading commensals in the large intestine. Nature.

[53] Caminero A. (2014). Diversity of the cultivable human gut microbiome involved in gluten metabolism: isolation of microorganisms with potential interest for coeliac disease. FEMS Microbiol Ecol.

[54] Carroll I.M. (2013). Fecal protease activity is associated with compositional alterations in the intestinal microbiota. PLoS ONE.

[55] Kastl A.J., Terry N.A., Wu G.D., Albenberg L.G. (2020). The structure and function of the human small intestinal microbiota: current understanding and future directions. Cell Mol Gastroenterol Hepatol.

[56] Klaus J. (2009). Small intestinal bacterial overgrowth mimicking acute flare as a pitfall in patients with Crohn’s disease. BMC Gastroenterol.

[57] Bouhnik Y. (1999). Bacterial populations contaminating the upper gut in patients with small intestinal bacterial overgrowth syndrome. Am J Gastroenterol.

[58] Vantrappen G., Janssens J., Hellemans J., Ghoos Y. (1977). The interdigestive motor complex of normal subjects and patients with bacterial overgrowth of the small intestine. J Clin Investig.

[59] Nieuwenhuijs V.B. (1998). The role of interdigestive small bowel motility in the regulation of gut microflora, bacterial overgrowth, and bacterial translocation in rats. Ann Surg.

[60] Suri J., Kataria R., Malik Z., Parkman H.P., Schey R. (2018). Elevated methane levels in small intestinal bacterial overgrowth suggests delayed small bowel and colonic transit. Medicine.

[61] Roland B.C. (2015). Small intestinal transit time is delayed in small intestinal bacterial overgrowth. J Clin Gastroenterol.

[62] Leinonen R., Sugawara H., Shumway M. (2011). on behalf of the International Nucleotide Sequence Database Collaboration. The sequence read archive. Nucleic Acids Res.

[63] Martin M. (2011). Cutadapt removes adapter sequences from high-throughput sequencing reads. EMBnet J.

[64] Callahan B.J. (2016). DADA2: High-resolution sample inference from Illumina amplicon data. Nat Methods.

[65] Almeida A. (2021). A unified catalog of 204,938 reference genomes from the human gut microbiome. Nat Biotechnol.

[66] Altschul S.F., Gish W., Miller W., Myers E.W., Lipman D.J. (1990). Basic local alignment search tool. J. Mol. Biol..

[67] Hyatt D. (2010). Prodigal: prokaryotic gene recognition and translation initiation site identification. BMC Bioinform.

[68] Rawlings N.D., Morton F.R. (2008). The MEROPS batch BLAST: a tool to detect peptidases and their non-peptidase homologues in a genome. Biochimie.

[69] Teufel F. (2022). SignalP 6.0 predicts all five types of signal peptides using protein language models. Nat Biotechnol.

[70] Waschina S., Zimmermann J., Kaleta C. (2023). gapseq reconstructions for 5414 genomes from the HRGM collection. Zenodo.

[71] Gelius-Dietrich G., Desouki A.A., Fritzemeier C.J., Lercher M. (2013). J. sybil – Efficient constraint-based modelling in R. BMC Syst Biol.

[72] Orth J.D., Thiele I., Palsson, B Ø. (2010). What is flux balance analysis?. Nat Biotechnol.

[73] Benjamini Y., Hochberg Y. (1995). Controlling the false discovery rate: a practical and powerful approach to multiple testing. J R Stat Soc Ser B (Methodol).

[74] Oksanen Jari (2022). vegan: community ecology package. R Package Version 2 6-2.

